# Predicting residue contacts using pragmatic correlated mutations method: reducing the false positives

**DOI:** 10.1186/1471-2105-7-503

**Published:** 2006-11-16

**Authors:** Petras J Kundrotas, Emil G Alexov

**Affiliations:** 1 Computational Biophysics and Bioinformatics, Department of Physics, Clemson University, Clemson, SC 29634, USA

## Abstract

**Background:**

Predicting residues' contacts using primary amino acid sequence alone is an important task that can guide 3D structure modeling and can verify the quality of the predicted 3D structures. The correlated mutations (CM) method serves as the most promising approach and it has been used to predict amino acids pairs that are distant in the primary sequence but form contacts in the native 3D structure of homologous proteins.

**Results:**

Here we report a new implementation of the CM method with an added set of selection rules (filters). The parameters of the algorithm were optimized against fifteen high resolution crystal structures with optimization criterion that maximized the confidentiality of the predictions. The optimization resulted in a true positive ratio (TPR) of 0.08 for the CM without filters and a TPR of 0.14 for the CM with filters. The protocol was further benchmarked against 65 high resolution structures that were not included in the optimization test. The benchmarking resulted in a TPR of 0.07 for the CM without filters and to a TPR of 0.09 for the CM with filters.

**Conclusion:**

Thus, the inclusion of selection rules resulted to an overall improvement of 30%. In addition, the pair-wise comparison of TPR for each protein without and with filters resulted in an average improvement of 1.7. The methodology was implemented into a web server  that is freely available to the public. The purpose of this implementation is to provide the 3D structure predictors with a tool that can help with ranking alternative models by satisfying the largest number of predicted contacts, as well as it can provide a confidence score for contacts in cases where structure is known.

## Background

The correlated mutations (CM) analysis has been used to predict pairs or networks of amino acids that are distant in the primary sequence but form contacts in the native 3D structure [1-5]. The basic presumption is that during evolution, proteins accumulate sequence variability due to spontaneous mutations. However, the variability within a family of proteins should not affect the protein fold and function. Thus, amino acid positions that are important for the fold and the function should evolve in an orchestrated manner to conserve both the fold and the function.

The CM method predicts contacting residues by analyzing the correlated variability of the amino acid composition at two or more positions within the multiple sequence alignment. Thus, the detection of homologous sequences and the generation of the multiple sequence alignment are crucial for the performance of the method. Both tasks require substitution rules and several approaches were explored: (a) the CM based on amino acid identity [6]; (b) the CM using substitution matrices [1] and (c) the CM with statistically delivered pairing potentials [7]. The last approach appears very promising; Vriend and coworkers [7] achieved 20% mean accuracy on a set of 118 non-redundant proteins taken from the HSSP database [8] using 6 Å distance cut-off. Another promising direction of predicting residue contacts is a combination of machine learning and the CM method. In a series of studies Casadio and co-workers applied neural networks in conjunction with the CM method to predict disulfide bridges [9] and contacting residues [10,11]. Applying their methodology to a set of 173 non-homologous proteins resulted in an average accuracy of 0.21 while automatic predictions on 29 targets of CASP3 with the CORNET [10] server resulted in an accuracy of 0.14. Recently, a novel neural network method [12] that utilizes sequence information, secondary structure and solvent accessibility predictions, and the overall properties of the entire protein was developed in the Rost lab. On a test set of 633 proteins the PROFcon server [12] achieved an accuracy from 0.1 to 0.4 depending on both the number of predicted pairs in respect to the query length and the sequence separation. To the best of our knowledge this is the best result reported yet, however, the method is quite computationally demanding. Rather than predicting pairs of contacting residues, the statistical coupling analysis (SCA method), developed in the Ranganathan lab was extensively used to predict networks of interacting residues that mediate allosteric transitions [5,13], functional specificity [14] or energetic connectivity [4]. The predictions were tested against the experiment and a very good agreement was reported [14,15].

Despite the apparent progress made in developing reliable methodology of predicting residues contacts, the accuracy of the current methods is between 0.1 and 0.4. At the same time, the CM generates many predictions with relatively good scores and thus provides a large pool of predictions that contains a significant fraction of the true contacts. Therefore, the problem lies not in improving the sensitivity of the methodology, but in improving the accuracy, e.g. in reduction of the false positive ratio. The main goal of this study is to suggest possible improvements in the confidence of the predictions and to make the predictions more protein-specific.

As previously mentioned, the correlated mutations method reveals possible residue pairs within a protein family. If the structural region is highly conserved within the family, it is most likely that no variation in the amino acid sequence will occur. In contrast, if the structural region is not well conserved within the family members, then the residue contacts most likely will not be preserved. We wish to elucidate a more specific series of predictions relative to a particular member of a given protein family. Therefore, in this study the predictions made for a whole protein family are subjected to a set of pairing rules with respect to the biophysical properties of the amino acid sequence of a protein of interest. To the best of our knowledge this is the first attempt to incorporate biophysically-related knowledge into the statistical methodology of the correlated mutations approach.

## Implementation

### Simplified Correlated Mutations (CM) method (a pragmatic approach)

The correlated mutations and statistical conservation analysis methods have been previously described in detail (see Refs. [1,3,5,7,16] for excellent description of the method). Our approach differs from the existing implementations of the CM methods, since we do not emphasize the scoring scheme of the algorithm and the details of theoretical formulation. Thus, we do not rank the predictions and do not specify how many contacts should be predicted. Instead of using the correlation score as usually done in the CM analysis (see Ref. [3] and references therein), we define a set of four parameters which are subjects of optimization.

**Figure 1:**
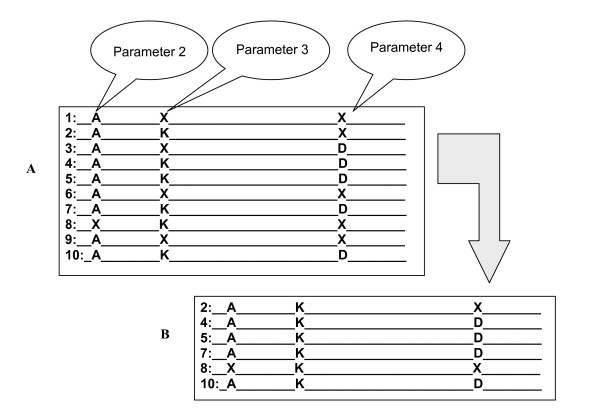
**Schematic representation of the correlated mutations algorithm**. The letter X stands for any residue not identical to the dominant residue in the corresponding position. The panel "A" illustrates multiple sequence alignment (MSA) and panel "B" shows sub-multiple sequence alignment (sMSA). The sMSA is obtained by selecting all sequence of the MSA having Lys residue at the position marked as Parameter 3.

The optimization is done on a set of known high-resolution structures so that the true prediction ratio (the number of true predictions divided by the total number of predictions) is maximized. The sequences of these test PDB files were submitted to the CM methodology to predict contacting residues. The predictions were then verified against the 3D structures. A prediction was considered to be correct (true positive) when any two heavy atoms of the side chains of the predicted residues were within 6 Å distance [7]. Predictions of contacts separated by less than six sequence positions (e.g. positions "i" and "i+s", s < 6) were considered trivial and omitted from the analysis. The optimization was done to maximize the true predictions ratio (TPR = true predictions/all predictions) by selecting the best values for several parameters (Figure 1) as described below.

### Purging the initial set of homologous sequences (parameter 1)

Each query sequence was subjected to a Ψ-Blast search against the database of non-redundant sequences obtained from the National Center for Biotechnology Information. The cut-off E-value remained the default (E-value = 10), but the number of output hits was increased to 2000 to ensure a sufficiently large pool of homologues. The resulting hits were then purged to remove short alignments (coverage less than 60% of the query sequence), very similar hits with sequence identities > 90% and very dissimilar hits with sequence identities < 20%. This approach differs from previous CM approaches because we remove "bad" sequence prior to performing the CM analysis. Now, the "bad" sequences will not affect the quality of the multiple sequence alignment which is crucial for the CM performance. The remaining homologous sequences were further purged with CD-hit [17] to remove homologous sequences at certain levels. This is similar to applying a correction coefficient to the correlation formula to downweigh information from very similar sequences (see eq.(1) in Ref. [3]). The level of CD-hit purging was considered the first parameter of the optimization, with a range variance of 90% to 40%. In addition, the cases that returned less than 20 homologues amino acid sequences were regarded as having little information and were subsequently deleted from the protocol.

### Excluding totally conserved residues (parameter 2)

The purged sequences were further subjected to CLUSTAL W [18] to generate multiple sequence alignment. Some of the positions of the multiple alignment were totally conserved while others showed little variation. A totally conserved position does not provide information necessary for the CM analysis and thus is deleted in our implementation. However, to reduce the noise from the occasional insertion of sequences dissimilar to the rest of the sequences in the multiple sequence alignment, the definition of "totally conserved position" was slightly relaxed. This second adjustable parameter in our approach was varied from 100% to 80%. Figure 1 illustrates this parameter on an example of 10 sequences where Ala residue in the position marked as "parameter 2" is presented in 9 sequences. Therefore if parameter 2 is set to 90 % or less, this site will be omitted from the analysis. The fact that the residue is not conserved in the remaining sequence indicates that most likely the 10^th ^sequence is not a member of this particular family.

### Definition of partially conserved position (parameter 3)

In our implementation of the CM method, we first searched the multiple sequence alignment (MSA) for partially conserved positions. The minimum degree of conservation was considered to be the third adjustable parameter in our approach and was varied from 90% to 40%. This position (hereafter referred to as position I) is then used to extract a sub-multiple sequence alignment (sMSA), in which this position becomes totally conserved. For instance, as shown in Fig. 1, if a position in a multiple sequence alignment has Lys residue within 6 out of 10 sequences, the degree of conservation is 60% (see Figure 1) and thus this position will be considered further if the parameter 3 is set to 60% or less. Removing all sequences not containing Lys residue in this position will result in a sub-multiple alignment that is shown separately in Figure 1. Similar sMSA's are constructed for all MSA positions that satisfy parameter 3.

### Finding correlated positions (parameter 4)

We then performed a second search within each sMSA (see previous subsection) to find partially conserved positions (correlated positions or position II). If in a particular sMSA position the given residue has a degree of conservation larger than a certain threshold, this position is considered to be correlated with the position I for which the sMSA was constructed. This minimum degree of conservation for the position II in this second search was considered to be the fourth adjustable parameter in our approach and was varied from 100% to 40%. An example of such position is shown in Figure 1 as a position with Asp residue in 4 of the 6 sMSA sequences resulting to a degree of conservation of 66%.

### Filters for reduction of the false positive ratio

The predictions made by the CM were subjected to a set of rules (filters) to filter out pairs that do not have complementary physical-chemical properties. The selection rules are introduced using general biophysical considerations and are not related to the pairing frequencies or residue pairing preferences delivered from statistical studies of residue contacts in the existing 3D structures. Residue contacts observed in protein structures may not reflect true residue interactions but rather could be caused by other factors. For example, the statistically observed large number of hydrophobic – hydrophilic residues' contacts perhaps reflects the overall protein structure made of a hydrophobic core surrounded by a hydrophilic shell of residues. Thus, we believe that using biophysically-based constraints better accounts for the driving mechanism of correlated mutations. In addition, the CM method is family specific, i.e. the predictions are based on the multiple sequence alignment of the entire protein family. Thus, it is possible that a prediction made for the entire family may not be suitable for the particular query.

The filters are based on the general considerations of the nature of the interactions between amino acids and thus, strictly speaking, are neither delivered statistically nor analytically, although small corrections in the original assumption were made during the optimization phase against the first set of proteins. Four physical-chemical characteristics were applied: hydrophobicity, polarity of the charge, hydrogen donor/acceptor pairing and disulfide bridge formation. Thus, two residues are considered to be in energetically favorable contact if they can form:

(a) a hydrophobic pair, i.e. when both residues in the predicted pair are hydrophobic ones (we included Trp in the list of the hydrophobic residues).

(b) an ion pair, i.e. when the residues within the predicted pair have opposite charges (His was included in the list of charged residues).

(c) a disulfide bridge (two Cys residues in the predicted pair).

(d) a hydrogen bond, i.e. hydrogen donor-acceptor pairs, like Asn and Gln paired with Asp, Glu, His, Lys and Arg.

(e) pairs, in which donation of a hydrogen bond to a hydrogen acceptor is possible, such as Ser, Thr or Tyr coupled to Asp or Glu.

Thus, if a predicted pair falls within one of the above categories, the pair is accepted, otherwise the prediction is deleted. In addition, during the optimization of the parameters, it was found that Gly residue quite often forms contacts with another Gly, and therefore Gly-Gly rule was also included in the list of acceptable pairs. At the same time very few true contacts were observed for Trp-Phe, Phe-Ile, Trp-Leu, Trp-Pro Pro-Phe, Pro-Ile, Pro-Leu, Pro-Met and Pro-Trp pairs and thus those pairs were excluded from the list of acceptable predictions.

**Figure 2:**
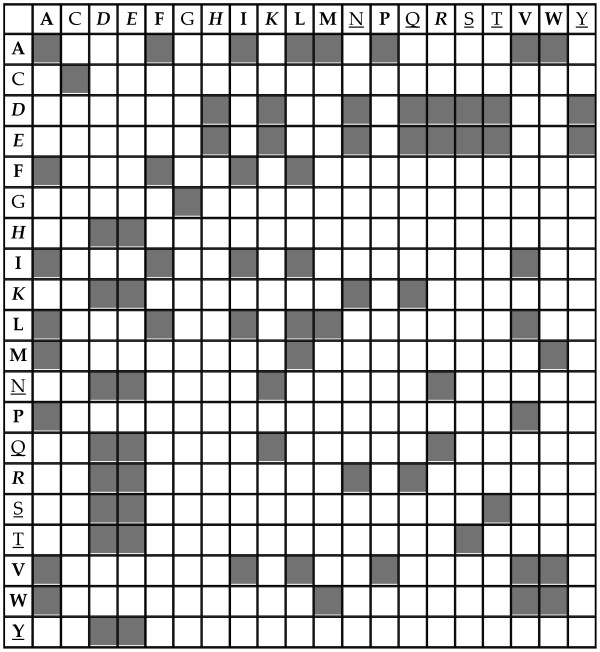
**Selection rules for predicted pair of residues**. Filled squares represent pairs that are allowed. The hydrophobic residues are in bold, hydrophilic residues in italic, hydrogen donor/acceptors are underlined.

It should be emphasized that these selection rules are applied in respect to the query sequence independently of the multiple sequence alignment. Thus, the predictions after the filters are query-specific. The selection rules are shown in Figure 2. Filled squares correspond to acceptable pairs, while empty squares correspond to pairs that are rejected. For convenience we refer to this methodology as Correlated Mutations with Filters (CMF).

### Web server

The RECON (REsidue CONtacts) web based server is intended to provide the scientific community with a publicly available tool capable of predicting intra-residue contacts by the correlated mutation method as described above. The front page of the server provides the user with options to either upload files with a sequence of interest to the server or to paste a sequence into the input window. Although the values of the four parameters are initially set to the optimum values reported in this paper, the user has the option to select other values from the list (with the 5% increments). Selection from the list rather than from the text fields is implemented in the server for convenience (an inexperienced user does not have to spend time understanding the appropriate range of the parameters), reliability (to ensure an appropriate format of the input data) and security (to reduce the number of manually input data). Users can also select whether biophysical constraints will be used during the calculations. Help buttons located at each selectable parameter and input field activate pop-up windows with a detailed explanation of the meaning of the corresponding parameters or input fields.

After the "Submit query" button is pressed, the home-made CGI Perl program controls the correctness of the input sequence. It will either verify that the submitted file is in the FASTA format or that the sequence contains the appropriate letters (20 amino acids name in the one-letter format plus letter X for an unknown residue). If mistakes are discovered, a pop-up window appears informing the user of the error. This popup also contains instructions for correcting the mistake made, so the user may resubmit the sequence.

If the submitted input is error-free, the program then displays information on the progress and completion of each calculation step. After simulations are completed, a new window appears containing the submitted sequence and the list of residue pairs predicted to interact. The residues are colored according to their physical-chemical classification (hydrophobic, polar, basic, acidic groups). A small embedded visualization script makes it easy to comprehend where in the sequence the predicted residues are located. When a user clicks on the predicted pair it gets highlighted both in the predicted contact list and in the displayed sequence. The program also offers the user an option to download the prediction results in a plain text file format.

### PDB files used for testing

The optimization of both CM and CMF protocols was accomplished using a set of high resolution structures. They were chosen using the Dunbrack cutting utility [19] applying two selection criteria (resolution smaller than 0.9 Å and sequence identity less than 20%). These criteria assure that the structures are of high quality and that they provide a diverse set of test cases in terms of their sequences. This selection yielded 29 structures (as of November, 2005). In 14 cases, the Ψ-Blast search against the non redundant database of sequences revealed less than the required 20 homologous sequences. Thus, the number of sequences/structures was dropped to 15. It can be argued that a training set of 15 structures may be too small to be representative of contact space. However, the set provided 9619 contacts in total, which is satisfactory from a statistical point of view. We refer to this set as the first dataset.

The second test was performed on a larger set of PDB structures obtained again from the Dunbrack server using a resolution criterion of 1.1 Å. The files from the previous test were excluded. This resulted in 137 files. However, not all sequences generated enough homologues in the Ψ-Blast search, which reduced the final number of test cases to 65. We refer to this set as the second dataset.

## Results

### Optimizing the parameters

**Figure 3:**
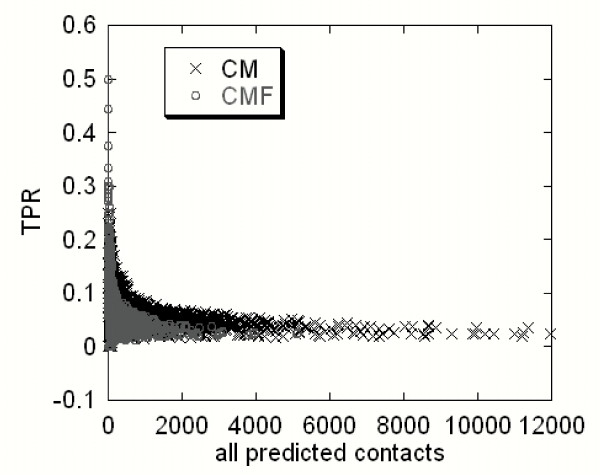
**True predictions ratio (TPR) as a function of all predicted contacts**. The predictions were made varying the values of all four adjustable parameters.

Each sequence of the first dataset was subjected to both CM and CMF algorithms. The predictions were made by varying the four adjustable parameters in increments of 0.1. Thus, for each sequence we generated many sets of predictions. The results, shown in Figure 3, show the peak of the distribution for both CM and CMF at a true positive ratio (TPR) of approximately 10%. Two observations can be made from a comparison of the CM and CMF results. The CMF reduces the overall number of predictions by approximately 6 times, but keeps the predictions with high TPR (see the distribution at TPR > 0.5). This feature is the core of our approach.

**Figure 4:**
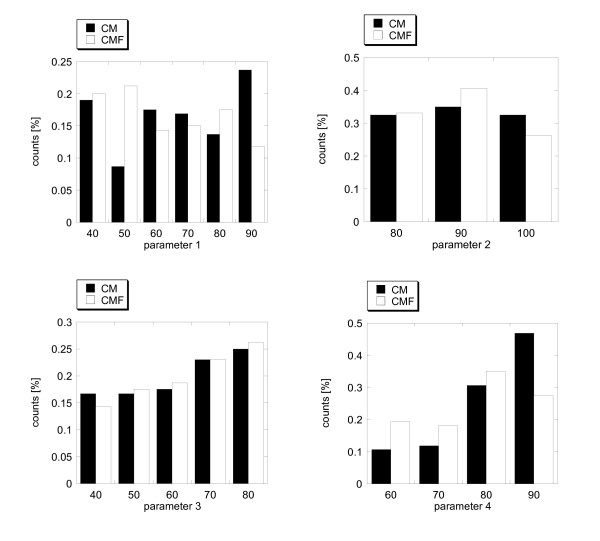
**The distribution of the adjustable parameters for the 10 best predictions for CM and CMF protocols**. Solid bars are the results of CM and striped bars are the results of CMF protocols. Ten best predictions were selected for each sequence in the dataset I from the pool of predictions for this sequence obtained with all possible combinations of parameter values used in calculations.

The above results were obtained by varying all four adjustable parameters. Since we do not score the predictions, we adopted the following strategy to find the optimal values of the adjustable parameters. Each query sequence generated a pool of predictions obtained by varying the adjustable parameters. From this pool we selected ten predictions with the best TPR for each sequence in the first dataset. The selection was done separately for the CM and CMF results. In certain cases, the TPRs were very good and were even equal to 1.0 (100% accuracy), but the predicted contacts were very few. To gain some statistical significance we neglected all cases that resulted to less than 6 predictions per query sequence. Thus, collecting 10 best results for all query sequences resulted in a pool of predictions that were used to count the frequencies of the values of the adjustable parameters (Figure 4). The maximal frequency among the best TPRs was found to occur at different parameter values for the CM and CMF. In the case of the CM, the optimal values for the four parameters were 0.9, 0.9, 0.8 and 0.9, respectively. In the case of CMF, they were 0.5, 0.9, 0.8 and 0.8, respectively.

### Benchmarking the first dataset

**Figure 5:**
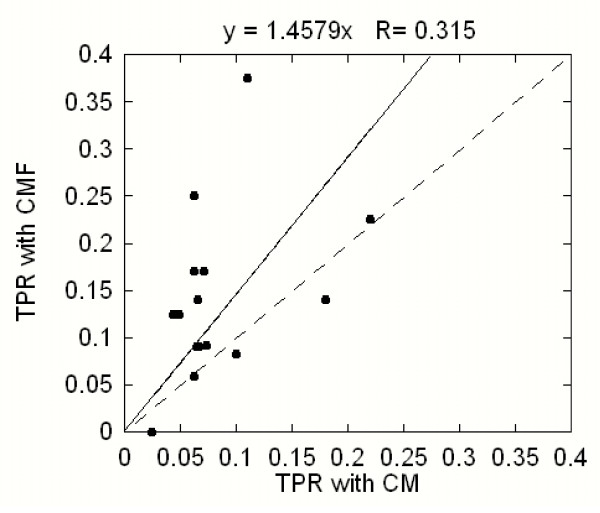
**True predictions ratio (TPR) of the correlated mutations protocol with biophysical filters plotted versus true prediction ratios of the correlated mutation protocol without the filters**. The solid line is the least-squares linear fit to the data points while the dashed line represents the diagonal. The results were obtained on the first dataset (see the text).

We again benchmarked the first dataset while keeping the parameters fixed at their optimal values. This resulted in a mean TPR of 0.14 for the CMF and a mean TPR of 0.08 for the CM protocols, indicating that the CMF outperformed the CM. The effect of selection rules is illustrated in Figure 5. Here we show the predicted TPRs of the CMF plotted against the TPRs of the CM for each sequence in the first dataset. The four parameters were kept fixed at their optimal values for the CMF obtained in the previous section. The results obtained with optimal values of the parameters for the CM are similar and thus are not reported. We applied the same cut-off as before requesting at least 6 predictions per query. In cases of less than 6 predictions, we performed sequential runs relaxing the fourth parameter until the number of predictions became larger than six. As can be seen, most of the points lie above the diagonal line demonstrating that the selection rules preferentially cut mostly false predictions, which in turn increases TPR. The least-square linear fit of the data points (Figure 5) results to a line slope of 1.5 indicating improvement of ~50% (ratio of TPR of the CMF versus TPR of the CM).

### Benchmarking the second dataset

**Figure 6:**
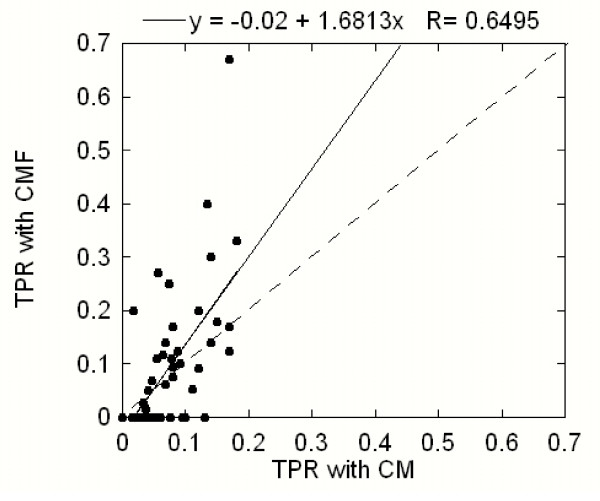
**True predictions ratio (TPR) of the correlated mutations protocol with biophysical filters plotted versus true prediction ratio of the correlated mutation protocol without the filters**. A solid line is the least-squares linear fit to the data points while a dashed line represents the diagonal. The results were obtained on the second dataset (see the text).

The above results are obtained on the same dataset that was used to obtain the optimal values for the four adjustable parameters. Therefore these results are considered biased and a further test was performed using the second dataset. Using the corresponding optimal values of the parameters for the CM and the CMF, we obtained a mean TPR of 0.09 for the CMF and a mean TPR of 0.07 for the CM. Though these results are less impressive than the results obtained from the first set, they still clearly indicate the improvement made by the selection rules, which on overall is 30% improvement of the mean value. The effect of the selection rules is demonstrated in Figure 6, which compares the TPR values of the CMF versus TPR values of the CM (note that data in Figure 6 were obtained using the optimal values of the CMF parameters for both the CMF and the CM calculations). The vast majority of the points again lie above the diagonal, which confirms that the selection rules selectively reject mostly false positives. The ratio between the TPR values of the CMF and the CM is now 1.7.

The length of query sequences may be a possible explanation for the less impressive results in the second dataset as compared with the first. The first dataset was generated at a resolution cut-off 0.9 Å and such high resolution structures are usually obtained for short-sequence proteins. Relaxing the resolution criterion to 1.1 Å, as done in the generation of the second dataset, longer query sequences were included. As repeatedly demonstrated, the CM performance degrades as the length of the protein increases. Indeed, analyzing the results obtained from the second dataset showed several outliers (very low TPR) corresponding to very long query sequences (longer than 600 amino acids). Thus, we would not recommend applying our method to predict contacts in sequences longer than 600 amino acids.

**Figure 7:**
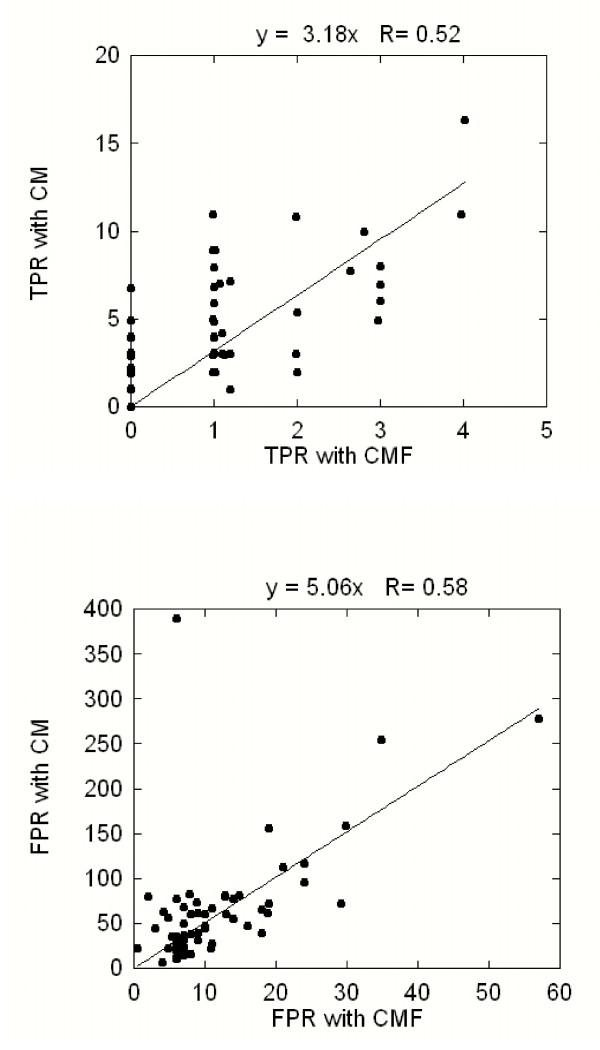
**Comparison of the effect of the filters on the true and false positives**. The upper panel shows the TRP of CMF versus the TRP obtained with CM methods. Applying filters reduces the TRP by an average factor of 3. The bottom panel shows the FPR of CMF versus the FPR calculated with CM protocols. The filters reduce the FPR by an overall factor of 5.

As it was mentioned in the introduction, the accuracy of the current CM methods varies between 0.1 and 0.4 depending on the number of predicted contacts in respect to the query length. Our implementation does not rank the predictions and thus it is impossible to control how many predictions will be made per query length. However, a plot of the number of the predictions for each query as a function of the query length (L) resulted to fitting line with a slope 1/4 for the predictions without filters and to 1/20 for the predictions with filters (data not shown). Thus, on average the CM protocol predicted L/4 pairs, while the CMF protocol made L/20 predictions. We would like to emphasize again that our protocol does not rank the predictions, and thus the reduction of the number of the predictions from an average of L/4 with the CM protocol to L/20 with the CMF protocol does not mean selecting the best L/20 predictions from the pool of L/4 predictions. If the filtering rules were randomly selected one should expect reduction of the number of predictions but no change of the TPR, since true positives and all predictions will be reduced by the same proportion. The selectivity of the filters is illustrated in Figure 7 where the true and false predictions are plotted for the CMF and the CM protocols, respectively. It can be seen from the slope of the fitting line that the filters reduce the true predictions made by the CM method by a factor of 3, while the false predictions are reduced by a factor of 5.

The main finding of the paper is that the biophysical filters always improve the quality of the predictions. The effect was tested against different versions of the sequence database, using different versions of Ψ-Blast and CLUSTAL W and the results were found to be consistent (data not shown). However, the individual predictions per protein were quite sensitive to the above factors. The reason for that is our simplified implementation of the CM analysis that uses cut-offs for the parameters rather than applying a scoring function to rank the predictions.

What could be the reason for the improvement introduced by the physico-chemical filters? Perhaps this is the combination of the statistical approach of the CM analysis in conjunction with the filters that makes the difference since the physical-chemical filters alone cannot make predictions. In many cases the CM analysis finds a correlation between two positions in the multiple sequence alignment, but these positions may be far apart in the 3D structure of the representative protein since a reason for the evolutionally related correlation would not necessarily be a physical contact. There could be other reasons of a different nature, for instance, functional cooperativity when the positions could be "connected" through rigid secondary structure elements. Thus, the CM predicts a pool of correlated positions such that some of them are contacting while some of them are not. Applying filters that require physical-chemical complementarity favors the fraction of the contacting positions and thus improves the TPR (since the benchmarking is in respect to the contacting positions).

Our method combines statistical and biophysical approaches. The statistical approach (CM method) is used to generate the initial predictions and then these predictions are filtered based on biophysical considerations (complementarity of the residues within the predicted pair). Although these two approaches are applied independently of each other, there certainly is an overlap since the statistics reflect, to some extent, the biophysical interactions between residues (for example among the native 3D structures of proteins). However, since the filters were not statistically delivered, we can not estimate this overlap.

## Conclusion

The main goal of this study was to maximize the confidence of the predictions of contacting residues in the new implementation of the correlated mutations method. The results presented in this paper show that a set of selection criteria based upon the physico-chemical properties of amino acids significantly improves the performance of the CM protocol. The improvement coefficient per protein was found to be 1.7 and overall improvement for the entire set of 65 proteins was 30%. Though the absolute value of the accuracy is not impressive (TPR = 0.09), we argue that the filters can be implemented into a more advanced CM method to improve the predictions (a work currently in progress). The method was implemented into a web server, freely available to the scientific community and which can be used for residue contact predictions needed in users research.

## Availability and requirements

The RECON Web server for predicting residue contacts using our implementation of the correlated mutation method is freely available.

Project home page: 

Operating systems: Internet Explorer on MS Windows and Mozilla browser on Linux systems.

Other requirements: Allowing pop-up windows and enabling Java in Internet browser

License: free

## Abbreviations

CM – Correlated Mutations without Filters

CMF – Correlated Mutations with Filters

TPR – True Positive Ratio (true predictions divided by all predictions)

FPR – False Positive Ratio (false predictions divided by all predictions)

PDB – Protein Databank

RECON – Residue Contacts

## Authors' contributions

EA and PK equally contributed in the development of methodology and to writing the manuscript. EA contributed to the development of the FORTRAN code for the correlated mutations and PK contributed to the development of the Web server. All authors read and approved the final manuscript.
